# Editorial: Epidemiological trends in hematological malignancies

**DOI:** 10.3389/fonc.2023.1151774

**Published:** 2023-02-14

**Authors:** Marcos de Lima, Jorge Castillo, Michele Merli, Valentin Garcia-Gutierrez

**Affiliations:** ^1^ Division of Hematology, The Ohio State University, Columbus, OH, United States; ^2^ Harvard School of Medicine, Dana–Farber Cancer Institute, Boston, MA, United States; ^3^ Division of Hematology, University Hospital Ospedale di Circolo e Fondazione Macchi - ASST Sette Laghi, University of Insubria, Varese, Italy; ^4^ Servicio de Hematología y Hemoterapia, Ramón y Cajal University Hospital, Madrid, Spain

**Keywords:** hematology, leukemia, multiple myeloma, epidemiology, prevalence

Ou et al. performed a global population-based epidemiologic study of disease burden of chronic lymphocytic leukemia (CLL) in years 1990 to 2019 that included disability-adjusted life years (DALY) and age-standardized rates (ASR). They observed that CLL age-standardized incidence rate is growing worldwide, except in North America and Oceania. Also, unsurprisingly, the socio-demographic index (SDI) correlated with the burden of death and DALY. SDI is a measurement of country/regions development status that correlates with health outcomes. In low SDI quintiles, age-standardized death rate and age-standardized DALY rates went up, while decreasing significantly in high SDI quintiles. Presumably this disparity reflects access to newer and expensive medications, and to health care in general. Unfortunately, information from low SDI countries is limited and CLL incidence may be underestimated. Despite this intrinsic constraint, the paper illustrates what physicians everywhere know firsthand: social economic inequality is a major determinant of survival for patients with cancer, a sad determinant that is depicted here in the context of CLL.

Tang et al. used the Surveillance, Epidemiology, and End Results (SEER) to describe epidemiologic, prognostic and treatment outcomes of patients with primary central nervous system lymphoma. The SEER Program is supported by the Unite States National Cancer Institute and provides information on USA cancer statistics (1). The authors showed that race, sex, age, marital status, surgical resection and chemotherapy were independently associated with survival. The paper reflects the power of large databases such as SEER, but also illustrates the limitations of such analyses. Detailed risk stratification and treatment aspects were not available, for example. But it is interesting to notice that single patients had worse survival than married patients, likely indicating the importance of support provided by a spouse caregiver. Patients identified as Black, despite representing only 7% of this cohort, also had shorter survival than White patients.

Wu et al. also used SEER to review survival trends in patients under age 65 years with mantle cell lymphoma (years 1995-2016). They divided years of diagnosis in three eras: chemotherapy-alone (1995-2000), intensified immunochemotherapy era (2001-2012) and targeted therapy era (2013-2016) and compared survival in these time frames. The patient population was predominantly white, but 5.8% were Black and 10.6% were Hispanic. In multi-variate analysis, male gender, age, and disease stage predicted survival, while race did not. Overall, survival improved significantly over the years, presumably a reflection of better treatments.

Neither Wu et al. or Tang et al. included ZIP code associated median income in their analyses. Others have showed marked inequality in disease outcomes by using this surrogate measurement of regional income (2). Chamoun et al. used the USA National Cancer Database to study socioeconomic factors in a large cohort of 117,926 patients diagnosed with multiple myeloma between years 2005 and 2014. Fewer comorbidities, younger age, treatment in academic centers, and living in a higher median income area were significantly linked with better survival, while race did not predict survival. A Veterans Affairs study showed that African Americans diagnosed with multiple myeloma had better survival than White patients when equal access to novel therapies and transplant was available (3). These analyses, flawed as they may be due to limited data nuances (4), point out that racial differences in outcomes are often complex surrogates reflecting socio-economic disparities rather than biologic factors.

The relevance of such studies will only increase as we see cancer treatments become more expensive and less accessible (5). The papers in this Research Topic bring important contributions to this debate.

## Author contributions

All authors listed have made a substantial, direct, and intellectual contribution to the work and approved it for publication.
